# The Relationship Between Childhood Physical and Sexual Abuse and Adolescent Cannabis Use: A Systematic Review

**DOI:** 10.3389/fpsyt.2021.631245

**Published:** 2021-05-26

**Authors:** Víctor De la Peña-Arteaga, Sarah O. Nogueira, Michael Lynskey, Lindsey A. Hines

**Affiliations:** ^1^Bellvitge Biomedical Research Institute, Barcelona, Spain; ^2^Faculty of Medicine and Health Sciences, University of Barcelona, Barcelona, Spain; ^3^Addictions Department, Institute of Psychiatry, Psychology & Neuroscience (IoPPN), King's College London, London, United Kingdom; ^4^Centre for Academic Mental Health, Population Health Sciences, University of Bristol, Bristol, United Kingdom

**Keywords:** childhood maltreatment, physical abuse, sexual abuse, adolescence, cannabis, drug use, systematic review

## Abstract

**Background:** Among adolescents, cannabis use is a health concern due to associations with drug addiction and mental health disorders across the life course. It has been shown that childhood maltreatment is associated with drug addiction in adulthood. However, a better understanding of the relationship between maltreatment and drug use may improve targeted prevention and interventions. The aim of this systematic review is to describe the association between exposure to childhood maltreatment, specifically physical and sexual abuse, with adolescent cannabis use.

**Methods:** A systematic search strategy was applied to Embase, PsycINFO, and Ovid MEDLINE(R) databases. Methods followed Preferred Reporting Items for Systematic Reviews and Meta-Analyses (PRISMA) guidelines. Abstract and title screening was performed to identify papers which reported an estimate of the association between childhood physical or sexual abuse and adolescent cannabis use. Full text screening of each paper was performed, and data were extracted and study quality assessed. Weighted means meta-analysis was performed on studies reporting odds ratios as effect estimates.

**Results:** Of 8,780 screened articles, 13 were identified for inclusion. Eight papers received a quality rating score indicating lower risk of bias. Eleven papers reported the relationship between childhood sexual abuse and adolescent cannabis use; effect estimates ranged from AOR 0.53–AOR 2.18 (weighted mean OR 1.29, 95% CI 1.08–1.49). The relationship between childhood physical abuse and adolescent cannabis use was reported in 7 papers; effect estimates ranged from AOR 1.25–AOR 1.87 (weighted mean OR 1.39, 95% CI 1.12–1.66). Differences in the strength of the evidence were observed by the method of exposure ascertainment, and there was some evidence of differences in association by gender, age of cannabis initiation, and the severity of the abuse.

**Conclusions:** This systematic review indicates childhood physical or sexual abuse may increase risk of adolescent-onset cannabis use. Few studies considered variation in timing of onset, or by gender. Adolescent cannabis use precedes is strongly associated with increased risk of negative mental health outcomes; further exploration of adolescent cannabis use's place on the causal pathway between childhood abuse and adult mental health problems is warranted to improve intervention.

## Introduction

Globally, cannabis is the most commonly used internationally regulated drug (1). Adolescence is a key period for initiation of cannabis use (2). Cannabis use in adolescence is considered an area of public health concern as adolescence is recognised as a key period for development (3, 4) and there is research associating drug abuse with neurobiological changes in the developing brain of adolescents (5). There are notable recent changes regarding cannabis; policy on its use is becoming more liberal worldwide (6), and cannabis use is increasing amongst young Europeans, with prevalence of past-month use amongst those aged 15–34 years estimated at 5.4% in 2017 (7).

Adolescent cannabis use is a key target for early intervention strategies (8). Recent reviews have identified that adolescent cannabis use raises likelihood of depression and suicide attempts in later life (9), and cannabis use is consistently associated with increased likelihood of psychosis (10). Additionally, adolescent cannabis use is associated with poorer education and employment outcomes (11–13), and with acute risks from use such as car accidents (14). In a stage-sequential model of drug use and addiction, initiation of drug use is a necessary stage before individuals can escalate in frequency of use and problematic use (15). Exploring the early stages of drug use can have implications for better understanding of the pathways that lead to adult addiction and mental health disorders. Consequently, there is value in identifying risk factors for adolescent cannabis use in order to target prevention and intervention efforts.

A known risk factor for addiction is childhood adversity. Meta-analyses have shown that experiencing Adverse Childhood Experiences (ACEs) can raise the likelihood of experiencing negative physical and mental health outcomes (16, 17). Recent systematic reviews and meta-analysis (18–20) have found evidence for an association between childhood maltreatment or past traumatic events and drug problems later in life. However, these reviews have focussed mainly on dependence across the life course. The authors are unaware of a review which has focussed exclusively on non-problematic cannabis use during adolescence.

Previous reviews have focussed on broad conceptualisations of childhood adversity and trauma, but it is not clear that all childhood ACEs (classically conceptualised as sexual, physical or emotional abuse, emotional neglect, substance abuse by the parents, parental mental illness or suicide attempt, violence between parents, parental separation, bullying and parental criminal conviction) have the same relationship to outcomes. In the present review, we explore childhood physical abuse (CPA) and childhood sexual abuse (CSA) as risk factors. It is estimated that, globally, half of all children will experience or witness some form of violence in childhood (21). Although global meta-analyses estimate the prevalence of physical and sexual abuse to be minimal to moderate severity (22), childhood physical and sexual abuse in childhood are both public health concerns given their association with negative outcomes across the life course (23). In relation to cannabis use and dependence, a study breaking down risk factors by stages of drug use found a relationship between CSA and exposure to cannabis, but not progression to dependence (24). Studies of these risk factors for illicit drug use have contradictory findings, with physical abuse more strongly associated than sexual abuse in some studies (25, 26), while others show the opposite relationship (27).

This systematic review focusses on general population studies to describe the association between exposure to the adverse childhood experiences of physical and sexual abuse and adolescent cannabis use. We explore the quality of the literature on the relationship between childhood physical and sexual abuse and discuss the consistency of findings and the implications for cannabis use.

## Methods

### Research Design

The present study consists of a systematic review of the literature based on the Preferred Reporting Items for Systematic Reviews and Meta-Analyses (PRISMA) guidelines.

### Information Sources

The electronic databases used for this systematic review were Embase, PsycINFO and Ovid MEDLINE(R). The search was performed at two different points in time: June 2017 and September 2020.

### Search

The following search terms were used to perform the search in the electronic databases [selected using a Participants, Interventions, Comparators, Outcomes, and Study design approach (28)]:

[(Teenage or Adolescent or Adolescence or Youth or Child) and (Maltreatment or “Child abuse” or “Sexual abuse” or “Physical abuse” or “Adverse experience” or Trauma or Stress) and (“Misuse” or “First use” or Initiation or “Illicit use” or “Use” or “Abuse” or Experimentation) and (Drug or “Illicit drug” or Cannabis or Marijuana or Hash^*^ or Skunk or Opiate or Heroin or Stimulant or Alcohol or Chemsex or “Novel Psychoactive Substance” or “Legal high” or Ecstasy or Cocaine or Meth or Tobacco or Nicotine or Cigarette)].tw.

The search was limited by title and abstract content (.tw.). No further limits were used. Alcohol, tobacco and other illegal drugs search terms were included to ensure capture of papers where cannabis use was a secondary focus.

### Eligibility Criteria

#### Inclusion/Exclusion Criteria

Studies were included if they met the following inclusion criteria:

1. Reported as part of a peer reviewed journal and as part of the databases used to the search or their references.
2. Individuals were up to 26 years old. This age range encompasses contemporary patterns of adolescent growth and their social role transitions (29), as well as capturing a period in which neuronal connexions are continuing to develop (30).
3. Papers published in English.
4. General population samples.
5. Included a measure of association between childhood physical or sexual abuse and cannabis use.

Studies were excluded if:

1. Reviews, meta-analysis, conference abstracts, dissertations, lectures, book chapters or incomplete articles.
2. Regarding sample: excluding
  - Individuals diagnosed with addiction or substance use disorder.
  - Animal studies, due to the aim of researching human adolescence.
  - Groups of drug using participants only or inpatients of an addiction clinic.

### Study Selection

Once the search was run, by two researchers (VDA and SON), results were exported into a reference manager software. Duplicates were removed using the same software and afterwards an abstract and title screening was performed to obtain the relevant full text studies. During this stage, papers were excluded using the criteria stated above.

Subsequently full text screening was performed by researcher review (VDA and SON) of each paper. Inclusion and exclusion criteria were applied as above to determine suitability for inclusion in review. Papers excluded at this stage mainly represented the ones outside of the age range or focused exclusively on cannabis use disorders rather than cannabis use.

As mentioned, the full search and screenings were performed by two researchers independently. Afterwards, each inconsistency was examined by another researcher (LH) to obtain a final list of included papers. To finalise, references of key papers were manually screened to ensure review completeness.

### Quality Assessment

An adapted version of the Newcastle-Ottawa Scale (NOS) for assessing the quality of non-randomised studies in meta-analyses [by Wells et al. (31)] was used for this review. With this tool, each study was judged on three broad perspectives: the selection of the study groups; the comparability of the groups; and the ascertainment of either the exposure or outcome of interest for case-control or cohort studies, respectively. Once again, papers were rated for quality by two researchers independently (VDA and SON) and inconsistencies were reviewed by a third researcher (LH).

### Weighted Mean Meta-Analysis

The studies reporting Odds Ratios (OR) and Confidence Intervals (CI) were entered into a weighted mean meta-analysis. Weights were assigned to each study taking into account each sample size and OR. This was obtained to summarise the global magnitude of effect sizes with the available data.

## Results

### Study Selection

**Figure 1:**
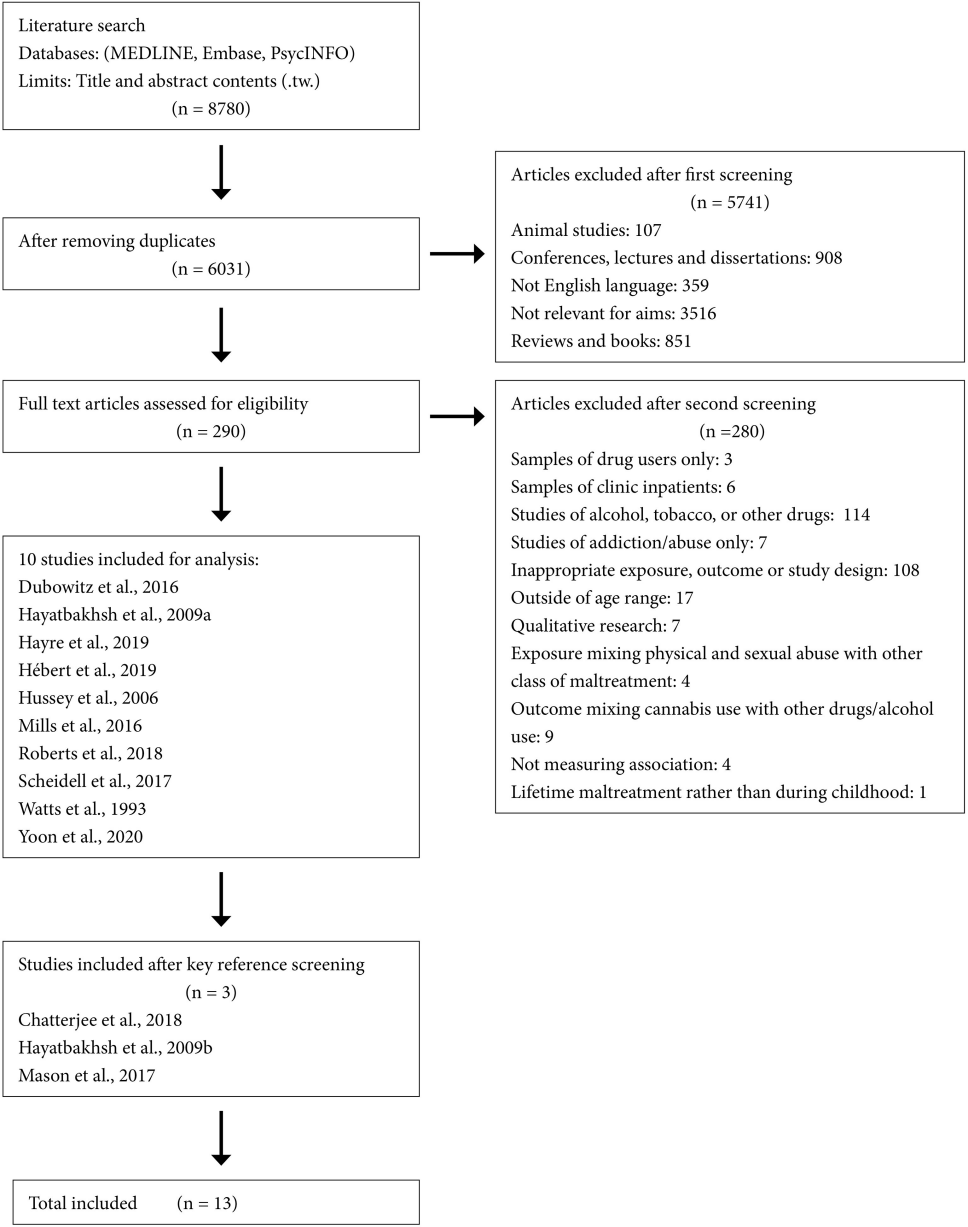
Flowchart of search results.

The final study selection of 13 papers is fully shown in Figure 1. Characteristics of each of the included studies can be seen in both quality assessment table (Table 1) and data extraction table (Table 2) (32–44).

**Table 1:** Quality rating.

**References**	**Representativeness of the cohort**	**Selection of participants who did not experience childhood abuse**	**Ascertainment of childhood abuse**	**Did data collection start before participants started using cannabis?**	**Did the study control for confounders that will make samples comparable?**	**Was follow-up long enough for outcomes to occur**	**Adequacy of sample retention**	**Total**
Chatterjee et al. (32)	1	1	0	0	2	0	0	4
Dubowitz et al. (33)	1	1	1	1	0	0	1	5
Hayatbakhsh et al. (34)	1	1	0	1	2	0	1	6
Hayatbakhsh et al. (35)	1	1	0	1	1	0	0	4
Hayre et al. (36)	1	1	0	0	1	0	1	4
Hébert et al. (37)	1	1	0	0	1	0	1	4
Hussey et al. (38)	1	1	1	1	2	0	1	7
Mason et al. (39)	1	1	1	1	1	1	1	7
Mills et al. (40)	1	1	1	1	2	0	1	7
Roberts et al. (41)	1	0	1	1	1	1	1	6
Scheidell et al. (42)	1	1	1	1	2	1	1	8
Watts et al. (43)	1	1	0	0	0	0	0	2
Yoon et al. (44)	1	1	1	1	0	0	1	5
***Selection***			Max 4*					
*Representativeness of the cohort.*			*truly/somewhat representative cohort sample; 0 selected group e.g., volunteers/no sampling description.					
*Selection of the participants who did not experience childhood abuse.*			*from the same community as the exposed cohort; 0 different source/no description.					
*Ascertainment of childhood abuse.*			*secure record (government record)/structured interview; 0 written self-report/no description.					
*Did data collection start before participants started using cannabis?*			*yes; 0 no.					
***Comparability***			Max 2*					
*Did the study control for confounders that will make samples comparable?*			*study controls for sex/gender; * study controls for SES.					
***Outcome***			Max 3*					
*Was follow-up long enough for outcomes to occur.*			*yes - followed up past age 25; 0 no. *complete follow up - all subjects accounted for.					
*Adequacy of sample retention.*			*subjects lost to follow up unlikely to introduce bias – > 60 % follow up, or description provided of those lost; 0 follow up rate <60% and no description of those lost/no description of retention or sample loss.					
Total maximum: 8*								

**Table 2:** Data extraction.

**References**	**Country**	**Study design**	**Measure of childhood abuse**	**% female**	**Mean age of sample (range)**	**Sample size**	**Cannabis use measure (lifetime, past year, frequency)**	**Statistical method**	**Univariable effect estimate (CI): composite measure of physical/sexual abuse**	**Univariable effect estimate (CI): physical abuse**	**Univariable effect estimate (CI): sexual abuse**	**Covariate adjustment**	**Adjusted effect estimate (CI): composite measure of physical/sexual abuse**	**Adjusted effect estimate (CI): physical abuse**	**Adjusted effect estimate (CI): sexual abuse**	**Notes**
Chatterjee et al. (32)	USA	Cross-sectional	Self-reported, semi-structured scale	49.7	9th, and 11th grade	79,339	First use age 14 and under	Multivariable logistic regression analysis	na	na	na	Race/ethnicity, poverty status, grade, family composition, school location, and connexions to parents	Female 1.24 (1.04–1.43) Male 1.21 (1.03–1.42)	na	na	
Dubowitz et al. (33)	USA	Prospective cohort study	Child protective services records	Not specified	18 and under	702	Some use	Multinomial logistic regression analysis	na	na	na	Peer use, neglect, emotional maltreatment, extent of childhood maltreatment, sex, site, ethnicity/race	na	1.25 (0.80–1.95)	1.52 (0.98–2.36)	
	As above	As above	As above	As above	As above	As above	Heavy Use	Multinomial logistic regression analysis	na	na	na	Peer use, neglect, emotional maltreatment, extent of childhood maltreatment, sex, site, ethnicity/race	na	1.23 (0.72–2.08)	0.80 (0.47–1.37)	
Hayatbakhsh et al. (34)	Australia	Prospective cohort study	Self-reported	52.1	21	3,285	Occasional use	Multinomial logistic regression	___	___	SA Once or twice 1.7 (1.2–2.3) SA 3+ times 2.5 (1.7–3.9) Raped 2.2 (1.4–3.3)	Gender, mother's age, mother's education measured at the child's birth, family income, marital status and quality mother-child communication measured at 14 years, maternal anxiety, depression, smoking, alcohol consumption measured at 14 years, child internalising and externalising measured at 14 years	___	___	SA Once or twice 1.4 (1.0–2.0) SA 3+ times 2.1 (1.4–3.3) Raped 1.6 (1.0–2.6)	
	As above	As above	As above	As above	As above	As above	Frequent use	As above	na	na	SA Once or twice 2.2 (1.5–3.5) SA 3+ times 3.3 (1.9–5.7) Raped 2.7 (1.6–4.7)	Gender, mother's age, mother's education measured at the child's birth, family income, marital status and quality mother-child communication measured at 14 years, maternal anxiety, depression, smoking, alcohol consumption measured at 14 years, child internalising and externalising measured at 14 years	na	na	SA Once or twice 2.8 (1.7–4.4) SA 3+ times 3.6 (2.0–6.4) Raped 3.1 (1.7–5.8)	
Hayatbakhsh et al. (35)	Australia	Longitudinal (MUSP), general population	Self-reported	34.3	21	3,754	Lifetime use	Multinomial logistic regression	na	na	Non-penetrative: 1.9 (1.4–2.5) Penetrative: 2.5 (1.8–3.5)	Gender, mother's age, changes in marital status, family income, problems in residential area, anxiety/depression, aggression/delinquency, nonverbal reasoning ability, school performance, puberty activity, child smoking, child alcohol use, TV watching, rule breaking at school, maternal smoking, maternal alcohol use, paternal history of crime, openness family communication	na	na	Non-penetrative: 1.7 (1.3–2.2) Penetrative: 1.8 (1.3–2.7)	
Hayre et al. (36)	Canada	Cross-sectional	Self-reported, semi-structured scale	59.3	12–18	528	Use in past month	Mediation sequential regression analysis	na	0.619 (0.245–0.993)	na	na	na	na	na	
Hébert et al. (37)	Canada	Cross-sectional school population	Self-reported, semi-structured scale	57.8	15.35 (grades 10–12)	8,194	Past year	Multivariable logistic regression analysis	na	na	na	Grade level, family structure, ethnicity. physical and emotional abuse, exposure to violence, exposure to interparental violence	na	na	Girls 2.18 (1.84–2.59) Boys 1.28 (0.88–1.84)	
Hussey et al. (38)		Prospective cohort study	Self-reported, structured, computerised	na	Grades 7–11	10,828	Last month	Binary logistic regression	___	1.65 *p* ≤ 0.001	1.76 *p* ≤ 0.001	Gender, age, race/ethnicity, parent's education, family income, immigrant generation, and US region	___	1.57 *p* ≤ 0.001	2.00 *p* ≤ 0.001	
Mason et al. (39)	US	Prospective cohort study	Interview, self-reported, childhood and adolescence	46	18	457	Life time	Path analysis	___	___	___	Mother's educational level, family after-tax income, mother's occupational level	___	___	r0.203 *p* < 0.01	
Mills et al. (40)	Australia	Prospective cohort study	State child protection agency records	52.6	21	3,778	Lifetime use	Logistic regression analysis	na	na	na	Age, gender, race, family income, maternal age, education, marital status, alcohol use, smoking, anxiety, depression	na	1.74 (0.91–3.34)	1.45 (0.77–2.72)	
	As above	As above	As above	As above	As above	As above	Early initiation	Logistic regression analysis	na	na	na	Age, gender, race, family income, maternal age, education, marital status, alcohol use, smoking, anxiety, depression	na	2.59 (1.37–4.89)	2.11 (1.13–3.94)	
	As above	As above	As above	As above	As above	As above	Daily use	Logistic regression analysis	na	na	na	Age, gender, race, family income, maternal age, education, marital status, alcohol use, smoking, anxiety, depression	na	2.94 (1.24–6.99)	3.08 (1.14–8.29)	
Roberts et al. (41)	US	Prospective cohort study	Child protective services records	55.6	18	847	Last month	Multilevel linear models	na	na	na	Gender, race, exposure to maltreatment	na	Beta: 0.06, SE: 0.14	Beta: −0.23, SE: 0.16	
Scheidell et al. (42)	USA	Prospective cohort study	Self-reported, structured, computerised	54.3	Age 11–21	12,288	Lifetime use	Logistic regression analysis	na	1.89 (1.60–2.24)	1.69 (1.39–2.05)	Each other type of trauma, age, gender, race, and poverty	na	1.38 (1.09–1.76)	1.29 (0.97–1.71)	Only took adolescent wave results, as the other ages fell outside of the review age range
Watts et al. (43)	US	Cross-sectional	Self-reported, non-structured	100	Grades 7–12	670	Lifetime use	Analysis of variance	na	na	r0.185	na	na	na	na	Results only presented for females, consequently analysis sample N 670
Yoon et al. (44)	USA	Prospective cohort study	Child protective services records	52.9	ages 12–18	903	Past year cannabis use	Binary logistic regression	na	na	na	Gender, race, household income	na	Early childhood abuse: 1.72 (0.95–3.10) Adolescent abuse: 1.87 (1.06–3.32)	Early childhood abuse: 0.55 (0.25–1.21) Middle childhood abuse: 0.53 (0.22–1.26)	

### Quality Assessment and Risk of Bias

According to the quality rating scale, one paper achieved the maximum rating of eight (42), and three got a very high grade of seven (38–40). A further eight papers received five or more of the available quality rating points and only one paper obtained a very low score of two (43).

Studies did not consistently differ by the representativeness of the cohort or the selection method of participants. Contrastingly, other rated categories, as control for confounders, ascertainment of childhood abuse, or data collection's start point were very different among studies. These differences created most of the variations seen in the final ratings. The interrater reliability between researchers was 0.42 (Kappa value).

### Definition of Physical and Sexual Abuse

Nine out of 13 papers used non-structured and structured self-reported scales (Table 2). Only one of these papers (39) included interviews as part of the assessment. The remaining four papers (33, 40, 41, 44) used data from child protective services records.

Three studies (34, 35, 44) differentiated outcomes based on the number or type of traumatic events. One study (34) made a differentiation between being sexually abused once or twice, three or more times and raped. When comparing frequency of events in adjusted models; occasional cannabis use Adjusted Odds Ratios (AOR) was 1.4 (95% CI 1.0–2.0) when sexual abuse was once or twice, compared to an AOR of 2.1 (95% CI 1.4–3.3) when sexual abuse was experienced three or more times. In contrast, frequent cannabis use AOR was 2.8 (95% CI 1.7–4.4) when sexual abuse was once or twice compared to an AOR of 3.6 (95% CI 2.0–6.4) when sexual abuse was experienced three or more times.

Additionally, one study (35) explored differences between the type of sexual abuse, comparing non-penetrative or penetrative. It did not identify differences for type of sexual abuse (35), finding an effect of AOR 1.7 (95% CI 1.3–2.2) for non-penetrative sexual abuse on any cannabis use, compared to AOR 1.8 (95% CI 1.3–2.7) for penetrative sexual abuse on any cannabis use.

One study (44) differentiated between timing of abuse for the association with cannabis use. Odds of recent cannabis use aged 12–18 were increased amongst those reporting adolescent physical abuse (AOR 1.87, 95% CI 1.06–3.32), but were not significantly different among those reporting early childhood physical abuse (AOR 1.72, 95% CI 0.95–3.10), early childhood sexual abuse (AOR 0.55, 95% CI 0.25–1.21) and middle childhood sexual abuse (AOR 0.53, 95% CI 0.22–1.26).

### Association Between Childhood Maltreatment and Adolescent Cannabis Use

For sexual abuse, a significant association between the exposure and adolescent cannabis use was observed in 5 of the 11 studies which focussed on this form of abuse (34, 35, 38, 39, 43), and 5 of the 11 studies reported weaker, non-significant evidence (33, 40–42, 44). One study (37) stratified by gender and found a significant association in females (OR 2.18, 95% CI 1.84–2.59), but a non-significant association for males (OR 1.28, 95% CI 0.88–1.84). Adjusted odds ratios ranged from AOR 1.4 (95% CI 1.0–2.0) (34) to AOR 2.00 (*P* ≤ 0.001) (38) in longitudinal studies, and as high as AOR 2.18 (95% CI 1.84–2.59) in a cross-sectional study (analysis restricted to females only) (37). Significant correlations of 0.19–0.2 between sexual abuse and lifetime adolescent cannabis use by age 18 were also reported (39, 43). In longitudinal studies the non-significant effect estimates ranged from AOR 0.53 (95% CI 0.22–1.26) (44) to AOR 1.52 (0.98–2.36) (33). Notably, all the studies reporting a significant association relied on self-report of sexual abuse from participants and the majority of the studies that identified weaker evidence used child protection records of childhood sexual abuse to determine the exposure (33, 40, 41, 44). Six studies reporting AOR and 95% CI (33–35, 40, 42, 44) were entered into a meta-analysis, producing a weighted mean effect of OR 1.29 (95% CI 1.08–1.49) for the relationship between childhood sexual abuse and adolescent cannabis use.

For physical abuse, a significant association between the exposure and adolescent cannabis use was observed in 4 of the 7 studies which focussed on this form of abuse (36, 38, 41, 42), and weaker evidence was reported in 2 of the 7 studies (33, 40). One study (44) differentiated timing of abuse, and consequently reported both significant and non-significant findings dependent on the timing of exposure (see discussion in section Definition of Physical and Sexual Abuse). Significant effect estimates ranged from AOR 1.38 (95% CI 1.09–1.76) (42) to AOR 1.87 (95% CI 1.06–3.32) when exposure to abuse was in early adolescence (44) in longitudinal studies. A Beta of 0.62 (95% CI 0.25–0.99) was reported in a cross-sectional study (36). This stronger evidence for an association between physical abuse and adolescent cannabis use came from studies with both self-reported abuse and child protection records as outcomes. All of the studies providing weaker, non-significant evidence were longitudinal, and used child protection records as their exposure. The non-significant effect estimates ranged from AOR 1.25 (95% CI 0.80–1.95) (33) to AOR 1.74 (95% CI 0.91–3.34) (40). Four studies reporting adjusted odds ratios and confidence intervals (33, 40, 42, 44) were entered into a meta-analysis, producing a weighted mean effect of OR 1.39 (95% CI 1.12–1.66) for the relationship between childhood physical abuse and adolescent cannabis use.

### Association Between Physical/Sexual Abuse and Age of Cannabis Initiation

Although not the primary focus of the review, some studies allowed an examination of the association between exposure to childhood maltreatment and age of onset of cannabis use. In a prospective cohort study, early onset of cannabis use (defined as prior to 17 years of age) was more than twice as likely amongst those who had experienced either physical abuse or sexual abuse (respectively, AOR 2.59, 95% CI 1.37–4.89 and AOR 2.11, 95% CI 1.13–3.94) (40). These effect estimates were significant, and stronger than for the association between either physical or sexual abuse and lifetime cannabis use (respectively, AOR 1.45, 95% CI 0.77–2.72 and AOR 1.45, 95% CI 0.77–2.72) (40). There was no clear linear pattern between sexual molestation and age of cannabis use in a cross-sectional study in U.S. school populations, with correlations reported by grade (7th−8th grade r0.19, 9th grade r0.03, 10th grade r0.15, 11th grade r0.09, 12th grade r0.35) (43), but it is notable that this study was rated low on quality.

### Gender Differences in the Relationship Between Childhood Abuse and Adolescent Cannabis Use

Two cross-sectional studies provided separate estimates of the relationship between childhood physical/sexual abuse and adolescent cannabis use for males and females. Experiencing physical or sexual childhood abuse was associated with a significantly increased likelihood of cannabis use at age 14 or under for both males and females (32). Effect estimates were similar across genders, with an AOR of 1.24 (95% CI 1.04–1.43) for females and AOR 1.21(95% CI 1.03–1.42) for males. Experiencing sexual abuse was significantly associated with over twice the likelihood of past-year cannabis use in Canadian school grades 10–12 for females (AOR 2.18, 95% CI 1.84–2.59), but with only a slight and non-significant increase in likelihood for males (AOR 1.28, 95% CI 0.88–1.84) (37).

## Discussion

This review identified 13 papers reporting an association between childhood physical or sexual abuse and adolescent cannabis use (defined in the search as use up to age 26) (29). There was good evidence for a relationship between both physical and sexual abuse in childhood and increased likelihood of adolescent cannabis use in studies where abuse was self-reported. The reported range of effect sizes was similar for both physical and sexual abuse, indicating that those who experience these forms of childhood abuse may be around twice as likely to report adolescent cannabis use. However, evidence was weaker in studies where abuse was determined using child protection records. There were more papers reporting associations for sexual abuse than for physical abuse, and most of the thirteen selected papers for this systematic review had a quality rating that indicated lower risks of bias.

The range of effect sizes was similar for the association between both physical and sexual abuse and adolescent cannabis use. However, of the papers that did report both measurements, those with better quality ratings for lifetime use (40, 42), showed a stronger effect size for the association with physical abuse in comparison with sexual abuse. Physical abuse has been identified as a risk factor for adolescent drug use (20) and subsequent transition to use disorders (45), but the present results demonstrate that it has received less focus in the literature than sexual abuse (reported in 7 studies, compared to 11 for sexual abuse).

The review identified differences in the strength of the evidence for the relationship between childhood physical/sexual abuse and adolescent cannabis use were related to the method of ascertainment of abuse. In this sense, studies with data from child protective services should be interpreted carefully. Although the reliability of this source is high, detection of exposed individuals may be lower due to unreported cases to authorities. As some researchers (40) have previously observed, “rates of retrospective self-report of child maltreatment are generally much higher than rates of agency confirmation, which raises the possibility of maltreated youth being misclassified.” Previous research has indicated that substantiated childhood maltreatment is no better at predicting outcomes than alleged (46). A recent review has identified that individuals who report abuse prospectively and retrospectively may represent different populations (47); consequently, it is important to compare differences between cross-sectional and prospective studies. In the present review cross-sectional and longitudinal designs were mostly in agreement, indicating that timing of reporting did not influence results.

Variations in exposure, such as the form and timing of the abuse, and the individual's gender may affect the relationship to adolescent cannabis use, but this is not widely explored in the literature. In the present review, one study (44) made a differentiation between early and middle childhood abuse, as well as adolescent abuse; only finding significant associations with past year cannabis use and physical adolescent abuse. Differences in the association with cannabis use were observed by frequency of the abuse and severity of the sexual abuse (34, 35). There were conflicting findings on whether effects may differ by gender (32, 40). Another recent review of this topic, focusing on cannabis abuse, also highlights the need for additional research on potential gender differences (19).

Adverse childhood experiences such as physical and sexual abuse are known to raise risks for life course negative mental health and addiction outcomes, and the present results indicate that adolescent cannabis use may be a plausible intervention target to mitigate these risks. A recent meta-analysis of the relationship between childhood physical/sexual abuse and adolescent cannabis abuse and dependence indicated that cannabis abuse/dependence is more likely amongst those experiencing physical abuse (OR: 1.58, 95% CI 1.01–2.46), and more than twice as likely amongst those experiencing sexual abuse (OR: 2.35, 95% CI 1.64–3.35) (19). Similarly, a comprehensive meta-analysis indicated likelihood of experiencing depression or anxiety was more than twice as high amongst those reporting childhood physical abuse (OR 2.00, 95% CI 1.25–3.19) or sexual abuse (OR 2.66, 95% CI 1.88–3.75) (48), and similar results have been found in relation to the likelihood of reporting psychotic experiences in a longitudinal cohort (physical abuse AOR, 2.24 95% CI, 1.75–2.87, sexual abuse AOR, 2.04 95% CI, 1.42–2.91) (49).

Adolescent cannabis use is a necessary step in the progression to the development of cannabis abuse/dependence (24), and is a commonly identified risk factor for anxiety, depression and psychosis (9, 50). The results of the present review add to the evidence that preventing adolescent cannabis use may be a viable intervention target for reducing risks of these negative outcomes amongst those experiencing early adversities such as physical and sexual abuse.

## Limitations

This review aimed to improve on previous studies by focussing the exposure to specific forms of adversity. However, a result of this approach was that we excluded studies which included composite measures of childhood abuse including non-physical abuses (e.g., emotional abuse and neglect). Future reviews may benefit from exploring the clustering of adversities. Our conclusions regarding sex differences and age of onset are weak considering the final number of studies that provided this information. To focus on cannabis use, distinct from abuse or dependence, studies of problematic use were excluded. However, this may have precluded us from identifying variation in frequency of use, which is an important consideration in the development of addiction and mental health. Studies did not commonly report unadjusted odds ratios, with the result that odds ratios included in the meta-analysis of weighted average have different adjustment patterns. A further limitation of the meta-analyses was the exclusion of studies that did not report odds ratios. Finally, we limited the search to studies published in the English language which may have excluded some relevant literature.

## Conclusions

There is some evidence both physical and sexual abuse may represent important risk factors for adolescent cannabis use. Adolescent cannabis use precedes the development of dependence, and is strongly associated with increased risk of negative mental health outcomes; further exploration of adolescent cannabis use's place on the causal pathway between childhood abuse and adult addiction and mental health problems is warranted to improve intervention.

## Data Availability Statement

The original contributions presented in the study are included in the article/supplementary material, further inquiries can be directed to the corresponding authors.

## Author Contributions

VD, LH, and ML developed the concept and scope of the paper. VD and LH produced the search strategy. VD and SN ran searches, screened papers, and completed quality rating and data extraction. LH oversaw these processes and resolved any conflicting decisions. VD drafted the manuscript, and LH performed meta-analyses. All authors made substantial contributions to the interpretation of the data and contributed to revising the manuscript critically. All authors approved the final version of the study to be published and are accountable for all aspects of the work.

## Conflict of Interest

The authors declare that the research was conducted in the absence of any commercial or financial relationships that could be construed as a potential conflict of interest.
